# Hydroxyapatite-Coated Sillicone Rubber Enhanced Cell Adhesion and It May Be through the Interaction of EF1β and γ-Actin

**DOI:** 10.1371/journal.pone.0111503

**Published:** 2014-11-11

**Authors:** Xiao-hua Shi, Shao-liang Wang, Yi-ming Zhang, Yi-cheng Wang, Zhi Yang, Xin Zhou, Ze-yuan Lei, Dong-li Fan

**Affiliations:** 1 Department of Plastic and Cosmetic Surgery, Xinqiao Hospital, the Third Military Medical University, Chongqing, 400037, People's Republic of China; 2 Department of Plastic and Cosmetic Surgery, Chongqing Armed Police Corps Hospital, Chongqing, 400061, People's Republic of China; 3 Department of War Trauma care, Hainan branch of PLA General Hospital, Sanya, Hainan, 572013, People's Republic of China; University of Akron, United States of America

## Abstract

Silicone rubber (SR) is a common soft tissue filler material used in plastic surgery. However, it presents a poor surface for cellular adhesion and suffers from poor biocompatibility. In contrast, hydroxyapatite (HA), a prominent component of animal bone and teeth, can promote improved cell compatibility, but HA is an unsuitable filler material because of the brittleness in mechanism. In this study, using a simple and economical method, two sizes of HA was applied to coat on SR to counteract the poor biocompatibility of SR. Surface and mechanical properties of SR and HA/SRs confirmed that coating with HA changes the surface topology and material properties. Analysis of cell proliferation and adhesion as well as measurement of the expression levels of adhesion related molecules indicated that HA-coated SR significantly increased cell compatibility. Furthermore, mass spectrometry proved that the biocompatibility improvement may be related to elongation factor 1-beta (EF1β)/γ-actin adjusted cytoskeletal rearrangement.

## Introduction

From its first use as an augmented rhinoplasty material in 1955, silicone rubber (SR) has become a common biological implantation material in plastic surgery [1]. According to clinicians, SR is easily processed into various shapes for medical devices, soft tissue filler, and even artificial organs [2], [3]. However, because of its compact structure and hydrophobicity, surrounding tissue cells cannot grow into the surface of the SR material, so it is easily to form capsule around the implant what is more to form capsular contracture [4]. The deposition of the collagenous structures onto the filler surface may lead to disfigurement as the implant bodies harden, shrink, or displaced, even greater harm, such as the implant bodies puncture the skin and lead to further complications including infection [5].

Hydroxyapatite (HA) is a naturally form of calcium apatite and is a key component of animal bones and teeth. By weight, it constitutes more than 96% of human teeth enamel and approximately 60% of human bone [6]. HA performs well in biocompatibility and corrosion resistance; furthermore, it has no toxic side effects. It can also integrates with the surrounding tissue and avoid the occurrence of rejection. For instance, when used for implantation in bone reconstruction, HA exhibits biocompatible properties but as a bulk material it suffers limitation because of its brittleness [7]. To counteract these difficulties, HA has been coated on to the surface of metallic implants, e.g. Citeau, Thian, Nelea, etc. [8]–[10] tried to blasting HA onto Titanium alloy (Ti6Al4V) and Magnetron *et al*. metallic materials found that HA coating can acquire both the mechanism properties and biocompatibility. The coating process can be made via several different methods including electro deposition plasma spraying, sputtering, and laser ablation *etc*. [11]–[13]. When implanted into bodies, the HA coating can help avoid the occurrence of rejection and encourages the integration of the implant with the surrounding tissue [14]. Thus, the implant is able to acquire both the desired mechanical properties and the required biocompatibility when HA is coated onto the surface of metal or other medical implants.

When HA was coated on to the surface of Ti6Al4 V, the material showed better bioabsorbability [15]. Using CoBlast to deposition HA onto a titanium substrate, Dunne *et al*. found a significant change to the surface properties of titanium [16]. Similarly, for nanostructured hydroxyapatite (nHA)/poly (lactic-co-glycolic acid) (PLGA) composite coatings on Mg-based substrates, the researchers observed synergistic properties that controlled the degradation of Mg-based substrates and improved bone-implant integration [17]. In addition to coating metal materials, HA is also used in composite with several types of polymers. For example, when HA was coated onto the surface of Poly L-lactic acid (PLLA) micro-fibers, that were implanted into Beagle dogs, histological and radiographic analysis showed that HA/PLLA screws induced significant increases in HA bone content from 36 months onward, and a burr hole was closed by 60 months [18].

In this study, we applied the HA coating process to SR, a material highly suitable for soft tissue implants, and measured the altered mechanical and cellular interaction properties of the SR. Our data indicate that we successfully produced HA coated SR materials, and the coating materials showed improved biocompatibility. Moreover, we developed a useful process for its production. In addition, through mass spectra we studied the molecular mechanism of cell adhesion.

## Materials and Methods

### Preparation of SR and HA coated SR

At temperature of 20°Cand humidity of 50%, a mixture of equal proportions (A∶B = 1∶1) of the two-component liquid SR (Chenguang Research Institute of Chemical Engineering, Chengdu, China) was slowly injected into a metal plate mold (100 mm ×100 mm ×2 mm) and placed into a vacuum chamber at −0.1 MPa for 30 min, then, cure at room temperature for 5.5 h. To coat the SR with HA, using custom-made spray painting equipment [19] with two sizes of HA (particle diameter of 40 µm, named HA-1, and particle diameter of 100 µm, named HA-2). The HA (National Engineering Research Center for Biomaterials, Sichuan University, China) was spread onto the surface of the SR mold after 5 h temperature curing, and it was left to cure for 0.5 h.

### Surface Characterization of HA coated SR

For SEM (scanning electron microscope, AMRAY 1000-B, Amray Inc, Bedford, Mass, USA) observations, SR and HA-SRs were cut into 10 mm×10 mm squares and dried in 37°C. Before observation, these squares were put into a vacuum pump to spray painting gold coat on the surface. X-ray photoelectron spectroscopy (XPS) was conducted on a Physical Electronics PHI 5802 equipped with a monochromatic Al Kα source to determine the surface chemical composition and elemental depth profiles. The sputtering rate was estimated to be approximately 5.67 nm•min^−1^ based on that calculated from a SiO_2_ standard sputtered under similar conditions, and the binding energies were referenced to the C 1s line at 285.0 eV. For Fourier transform infrared spectroscopy (FTIR) each material was cut into 10 mm ×10 mm squares and cleaned with dehydrated alcohol. After the alcohol volatilized, the material surface composition was studied by FTIR (Ni-colet 470 spectrometer); wave number is 4 cm^−1^; the scan extent is 4000∼400 cm^−1^. The water contact angles of SR and two kinds of HA/SRs were measured with a drop shape analysis system (DSA100, Krü ss). Test was in the sessile mode at room temperature. The roughness of the three kinds of SR was tested by LEXT OLS4100 laser confocal microscope (Olympus, Janpan), using 20x objective and the scan size is 13352 µm, each sample repeats 6 times.

### Mechanical Properties

**Figure 1:**
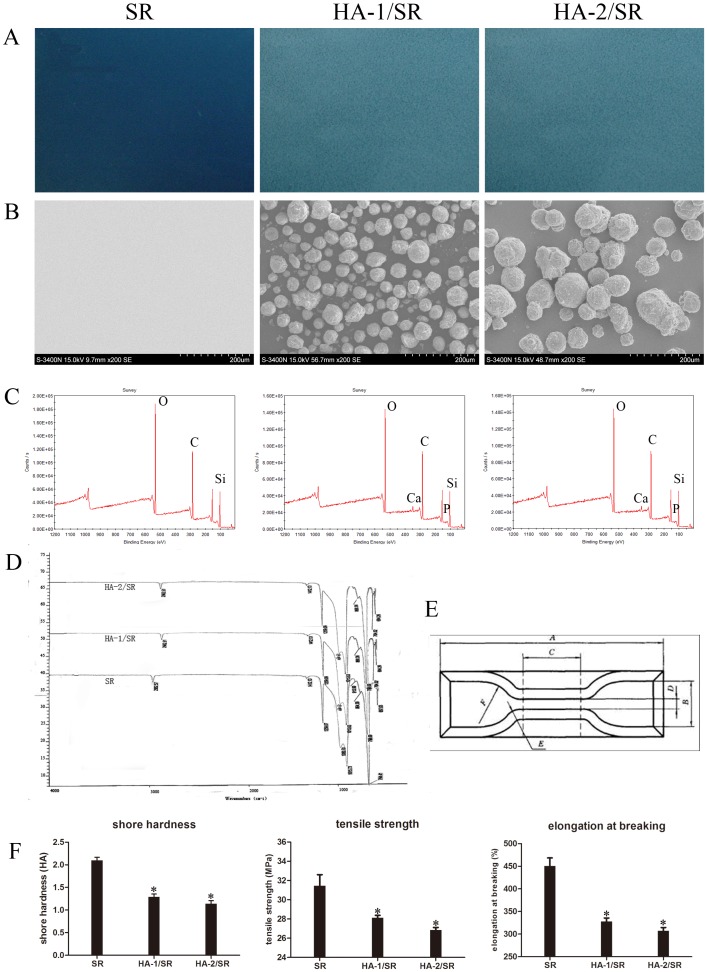
Characterization of HA coated SR. A, Optical microscopy pictures of the three kinds of samples (Left, SR; Middle, HA-1/SR; Right, HA-2/SR); B, SEM pictures of the three samples(Left, SR; Middle, HA-1/SR; Right, HA-2/SR); C, X-ray photoelectron spectroscopy of the three samples; D, FTIR of the three samples; E, Dumbbell shape of the sample for the mechanical properties study; F, Mechanical properties of the three samples (Shore hardness; elongation at breaking; tensile stress). Each number has six repeat, the average and stander standard error of the mean was used.

An A-Type Shore hardness meter (Harbin Measuring & Cutting Tool Group Co. Ltd, China) was used to test the Shore hardness of the coated and non-coated SR. The samples were soaked in 75% medical alcohol for 3 min, then sonicated for 15 min in deionized water, and dried at 50°C. Three pieces of overlapping samples in thickness of 5 mm were placed on a smooth, flat metal plate to test the Shore hardness. The spacing between the two test points was greater than 6 mm, and the distance from the test point to the sample edge was 20 mm. Each sample test more than six times. In order to detect tensile stress-strain properties, vulcanized SR were cut into a dumbbell shape (Figure 1E), fixed on an electronic universal testing machine (Exceed, E44 MTS, USA). Following three times of pre-stretching at a speed of 30 mm/min and an intensity of less than 2 N, measurements were taken of tensile strength and elongation until breakage.

### Cell Culture

Dermis fibroblasts (Homo sapiens) were employed to investigate the effects of HA coated SR on cell behavior. All cells were cultivated in a complete cell culture medium consisting of a mixture of Dulbecco's modified Eagle medium (DMEM, Gibco, USA) and 10% fetal bovine serum (FBS, Gibco, USA) in a humidified atmosphere of 5% CO_2_ at 37°C.

### Cell Adhesion and Cytotoxicity

Prior to cell cultivation, the samples were sterilized by immersion in 75% (v/v) ethanol for 30 min and subsequently rinsed three times with sterile phosphate buffered saline (PBS). The dermis fibroblasts were seeded on each sample in 24 well tissue culture plates at a density of 1×10^4^ cells per well and cultured for 24 h. Afterwards, the seeded samples were rinsed twice with sterile PBS, fixed with 4% polyoxymethylene solution, and stained with fluorescein isothiocyanate (FITC)-labeled actin tracker (Beyotime Institute of Biotechnology, China) sequentially. Cell adhesion was determined from three random fields using a fluorescence microscope (Leica TCS SP5, Germany). For cytotoxicity detection, propidium iodide (PI) and Hoechst 33342 double staining was used. The final concentration of Hoechst 33342 solution was 1µM, and the final concentration of PI was 10µg/ml. Cells were cultured on the material surface for 24 h, rinsed two times with PBS, and incubated with Hoechst 33342 at 37 °C in darkness for 15 min. The Hoechst 33342 solution was then removed with a PBS rinse, and PI dye was added and incubated at 4°C for 15 min in darkness, followed by PBS rinse. Laser scanning confocal microscope (Leica TCS SP5, Germany) was used to observe the staining. Hoechst 33342 krypton laser excitation with a UV fluorescence excitation wavelength of 352nm and an emission wavelength of 400∼500 nm, produces blue fluorescence. PI fluorescence with an argon ion laser excitation, excitation wavelength of 488 nm and emission wavelength greater than 630 nm, produces red fluorescence.

### Cell Proliferation Assay and Cell morphology observation by SEM

A Cell Count Kit-8 (CCK-8, Dojindo, Japan) assay was used to determine the cell viability and cell proliferation. Fibroblasts were seeded at a density of 5×10^3^ cells/well on the samples in 96-well tissue culture plates and cultured for 2 days. At the end of the incubation period, the samples were rinsed twice with sterile PBS and transferred to a new 96-well tissue culture plate. The attached cells were incubated in DMEM containing 5 mg/mL CCK-8 for 2 h. The optical density (OD) values were recorded by a Power Wave Microplate Spectrophotometer (Thermo, USA) at 450 nm to determine the cell viability. The cytoskeleton was stained with FITC-labeled actin Tracker probes (Beyotime Institute of Biotechnology, China). The results of the *in vitro* cell experiments were statistically analyzed using one-way analysis of variance (ANOVA) and a *p* value of less than 0.05 was considered to indicate statistical significance. As described previously [20], after cultured as monolayer on SR or HA/SR surface, human dermal fibroblasts were rinsed with PBS and fixed with 3% buffered glutaraldehyde for 20 min at 4°C. Then aqueous ethanol (30–100%) was used for dehydration step by step. Samples were lyophilized and coated with platinum. Cell morphology was observed by SEM (AMRAY 1000-B, Amray Inc, Bedford, Mass, USA).

### Detection of cell adhesion molecules

Western blot was used to detect the expression of adhesion related molecules with a mouse anti-human vinculin monoclonal IgG (Sigma-Aldrich, USA) at a dilution of 1∶1000. Horse radish peroxidase (HRP) labeled goat anti-mouse IgG (Santa Cruz, USA) was used at a dilution 1∶1000; rabbit anti-human zyxin polyclonal IgG (Millipore, USA) was diluted to 1∶1000, rabbit anti-human talin polyclonal IgG (Millipore, USA) diluted into 1∶1000, rabbit anti-human OPN polyclonal IgG (Santa Cruz, USA) was diluted to 1∶300, and HRP labeled goat anti-rabbit IgG (Santa Cruze, USA) was diluted to 1∶1000. Antibody binding was detected using an enhanced chemiluminescence (ECL) detection system (Advanstar, USA). The intensity of each blot was quantified by Quantity one software, and was normalized to the loading control (GAPDH). Each experiment was repeated at least three times. For immuno-fluorescence experiments, human dermal fibroblasts were cultured on SR or HA-SR surfaces as a monolayer were washed three times with PBS, and fixed in cold paraformaldehyde (4%) for 15 min at 4°C. Cells were then blocked with 5% bovine serum albumin in PBS (pH 7.5) for 30 min, followed by overnight incubation with the primary antibody as following: rabbit anti-talin (Abcam, USA, 1∶500), rabbit anti-zyxin (Cell Signaling, USA, 1∶500), rabbit anti-OPN (Santa Cruz, USA, 1∶100), or mouse anti-vinculin (Sigma-Aldrich, St. Louis, MO, USA, 1∶500). The corresponding Cy3 or FITC-tagged secondary antibody (Invitrogen, Shanghai, China) was then added, and incubated for 1h at room temperature. The cell nuclei were stained with 4′, 6′-diamidino-2-phenylindole (DAPI; 0.5 µg/ml; Sigma-Aldrich, St. Louis, MO, USA). Cells were visualized by using a Leica confocal microscope (Leica TCS SP5, Germany) with the appropriate filters. All measurements were repeated six times for each condition.

### Total RNA isolation and real-time reverse transcriptase polymerase chain reaction (RT-PCR)

The expression of talin, zyxin, OPN, vinculin mRNA was analyzed by real-time reverse transcription-polymerase chain reaction (RT-PCR), GAPDH mRNA expression was as control. Total RNA was prepared from cultured cells using TRIzol reagent (Invitrogen, CA, USA) according to the manufacturer's introduction. Spectrophotometrically at A260 and A280 were hired to measure the concentration and purity of RNA. ReverTra Ace RT-PCR kit (TOYOBO, Janpan) according to the manufacturer's instruction was used for RT-PCR. The resulting cDNA was used as a template for PCR with specific primer pairs using Primer Premier 5.0 software (Premier Biosoft, International, Palo Alto, CA, USA). The results were analyzed using delta Ct. All real-time PCRs were performed three times at least.

The primers used in the experiment are as bellows:

homo zyxin sense: GACCCAGGACCCAACAT,

homo zyxin antisense: CCTCCGCAAGCAGAGTA;

homo vinculin sense: ACAGATAAACGGATTAGAAC,

homo vinculin antisense: GCATTGTGAACCAGCA;

homo talin sense: CTGACAACAACCCTCAAC,

homo talin antisense: CCATTGGTCCTTCATCTA;

homo OPN sense: CAGCCAGGACTCCATT,

homo OPN antisense: TGTCAGGTCTGCGAAA;

homo GAPDH sense: ACCACAGTCCATGCCATCAC,

homo GAPDH antisense: TCCACCACCCTGTTGCTGTA.

### Mass Spectrometry Analyses

Total cellular protein was extracted from SR and HA-coated SR surfaces after 48 h of culture. The protein was extracted and separated in 8% SDS-PAGE and stained with Coomassie blue. Protein bands were cut out of the gel for analysis by mass spectrometry (VG Auto Spec 3000). The mass spectrometry results were then analyzed using the tools of the NCBI and EMBL databases.

### Generation of EF1β knockdown stable dermal fibroblasts by lentiviral infection and cytoskeleton stain

The dermal fibroblasts were seeded in a 6-well plate with 60% confluence in growth medium with polybrene,the EF1β knockdown stable dermal fibroblasts was constructed as before mentioned [20], using the EF1β shRNA lentiviral. The cytoskeleton was stained with FITC-labeled actin Tracker probes (Beyotime, Shanghai, China).

### Statistical analysis

The data presented in this study were expressed as means ± standard error of the mean (SE). Statistical differences were analyzed by one-way ANOVA followed by multiple comparisons performed with post hoc Bonferroni tests (SPSS version 16.0). The standard value of *p*<0.05 was considered statistically significant. The significance of any differences between two groups was tested using the paired-samples *t*-test, when appropriate.

## Results

### HA coated SR Preparation and Physicochemical properties

Optical microscopy shows that SR was colorless and transparent (Figure 1A). In contrast, large amounts of white HA particles were clearly visible on HA coated SR (Figure 1A). Additionally, SR surface was flat and smooth without any impurities, while the HA-coated SR surface is uneven with a large number of uniformly sized HA particles tightly adhered to the surface (Figure 1A). In addition, the water contact angle of HA-coated SRs decreased a little compare to SR, but not significantly (Table 1).

**Table 1:** Water contact angle of the three kinds of SR surface (n = 6, 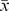
±s).

Group	SR	*HA-1/SR*	*HA-2/SR*
Water contact angle	111.4±2.7	107.7±1.1	106.3±1.5

SEM results showed that hydroxyapatite particles firm adhesion on the surface of SR (Figure 1B), the surface roughness of the material increased after hydroxyapatite coating, and HA/SR surface roughness also increased with hydroxyapatite particle size increase (Table 2).

**Table 2:** Surface roughness of the three kinds of SR surface (n = 6, 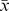
±s, * P<0.05).

Group	SR (µm)	HA-1/SR (µm)	HA-2/SR (µm)
Surface roughness	3.808±0.165	6.856±0.066*	8.094±0.1342*

The main components of SR are carbon, silicon, and oxygen (C, Si, and O). Thus, analysis of C, Si, O content with the HA modified surfaces can determine if there is any change in chemical composition. X-ray photoelectron spectroscopy (XPS) (Figure 1C, Table 3) showed that in HA-1/SR, there was a small amount of C reduction relative to the standard. XPS spectra of pure SR, C1s, Si2p, and O1s peak and peaks as well as of HA-1/SR and HA-2/SR showed a fluctuation range <0.1 eV, and they were not significantly different. While the corresponding calcium and phosphorus (Ca, P) corresponding peaks of HA-1/SR and HA-2/SR were observed (Table 3).

**Table 3:** Chemical composition (%) according to XPS analysis.

Group	%/binding energy) C	%/binding energy Si	%/binding energy O	%/binding energy Ca	%/binding energy P
SR	45.52/285.01	29.45/102.52	25.02/532.65		
HA-1/SR	43.86/285.02	28.33/102.55	26.33/532.7	0.77/348.19	0.71/133.94
HA-2/SR	42.65/285.01	27.46/102.7	24.54/532.65	2.69/347.91	2.61/134.26

FTIR (Figure 1D) measurements detected the following bonds: -CH3 anti-symmetric stretching vibration absorption peaks at 2962±1 cm^−1^; Si-O (-C) diffraction peak at 1080±1 cm^−1^; -OH bending vibration wave at 680±10 cm^−1^; diffraction peak of CO_3_^2−^ is at 1412±1 cm^−1^. In the spectra of HA-1/SR and HA-2/SR, HA was only sprayed onto the surface of SR, and thus its total content is very little, so the peak of PO_4_^3−^ at 950∼1200 cm^−1^ was not significantly different between HA-1/SR and HA-2/SR.

The mechanical property data (Figure 1F) show that compare to SR, HA-1/SR and HA-2/SR had statistically significant (*p*<0.05) differences in terms of Shore hardness, tensile strength, and elongation. While between HA-1/SR and HA-2/SR there was no statistically significant difference (*p*>0.05).

### Cell Compatibility

**Figure 2:**
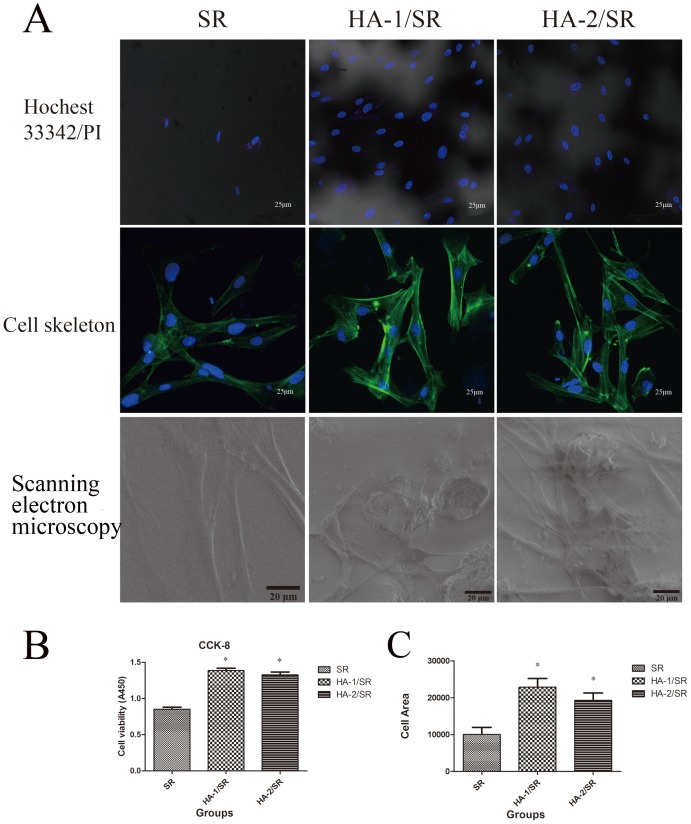
Cell compatibility of HA coated SR. A, Cytotoxicity of materials according to Hoechst 33342 double staining with PI (Left, SR; Middle, HA-1/SR; Right, HA-2/SR); Cell skeleton structure staining of each group (Left, SR; Middle, HA-1/SR; Right, HA-2/SR) (the scale bar is 25 µm); SEM images further demonstrated a more fibroblastic appearance of fibroblasts cultured on HA/SR (the scale bar is 20 µm); B, Cell proliferation of each group studied by CCK-8 (n = 6, *p<0.05); C, Cell area of each group studied by IPP6 (n = 6, *p<0.05).

As shown by the double staining of Hoechst 33342 with PI test for cytotoxicity of materials shown in Figure 2A, the cells suffered no significant toxic effects from either SR or HA/SRs. The results of the FITC-labeled actin-tracker, used to label actin and hence the cellular cytoskeletal structure, showed that in cells on the non-coated SR group, actin staining was less, the protrusion of edges was shrink, and filaments were sparsely arranged, compared to the HA-coated materials. In contrast, cells on the HA/SR surface showed greater green fluorescence, outward expansion of edge protrusions, greater filament increased, a rearranged cytoskeleton into fiber bundles, and fibroblasts cells with irregular long spindle protruding edges but with regularly arranged and evenly distributed filaments along the projections. The HA-1/SR surface had more obvious cytoskeletal rearrangement than that of HA-2/SR, suggesting that its surface is more conducive to cell adhesion (Figure 2A). Cell proliferation was detected with CCK-8 (Figure 2B). Both HA coated surfaces, showed OD values significantly higher than that of uncoated surface groups (*p*<0.05).

According to the evaluation standard of ISO 10993-5:1999 (Table 4), we determined the cytotoxicity of the material (Table 5), the relative growth rate (RGR) was calculated from the OD values using the following Calculation formula: RGR  =  (OD value of test sample group - OD value of blank)/(OD value of control group - OD value of blank) ×100%. According to the standard of ISO 1093-5:1999 and the people's Republic of China GB/T 16886.5-2003, in biological evaluation of medical devices in vitro cytotoxicity test, high density polyethylene used as a negative control reaction materials. Both the HA coated materials, HA-1/SR and HA-2/SR, had RGR values >100%, indicating a toxicity level of 0. The pure SR had an RGR value between 75% and 99%, indicating a toxicity level of 1 (Table 5). Therefore, HA coated SR has no toxic effects to cells and is in fact safer than SR.

**Table 4:** ISO 10993-5:1999 standard of cytotoxicity evaluation of biomaterials according to RGR.

Cytotoxicity level	RGR(%)
Level 0	≥100
Level 1	75–99
Level 2	50–74
Level 3	25–49
Level 4	1–24
Level 5	0

**Table 5:** Cell growth condition of SR and HA/SR (n = 6, 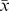
±s, * P<0.05).

group	OD value	RGR (%)	Cytotoxicity level
SR	0.8511±0.0191	76.39	Level 1
HA-1/SR	1.3877±0.0119*	138.86	Level 0
HA-2/SR	1.3280±0.0183*	131.91	Level 0

Cell proliferation analysis (Table 5, Figure 2B) showed that the proliferation of cells in the HA-1/SR and HA-2/SR groups showed cell growth greater than that of the SR group (*p*<0.05); while the SR group shows a slower cell growth rate with significantly fewer cells (*p*<0.05).

SEM image results further demonstrated a more fibroblastic appearance of fibroblasts cultured on HA/SRs (Figure 2A). We also quantified the morphology of fibroblasts on different substrates. The cell area depicted higher values for cells on HA coated SRs than those on SR (Figure 2C), But there was no statistically significant difference between HA-1/SR and HA-2/SR (*p*>0.05).

### Cell Adhesion Molecules

**Figure 3:**
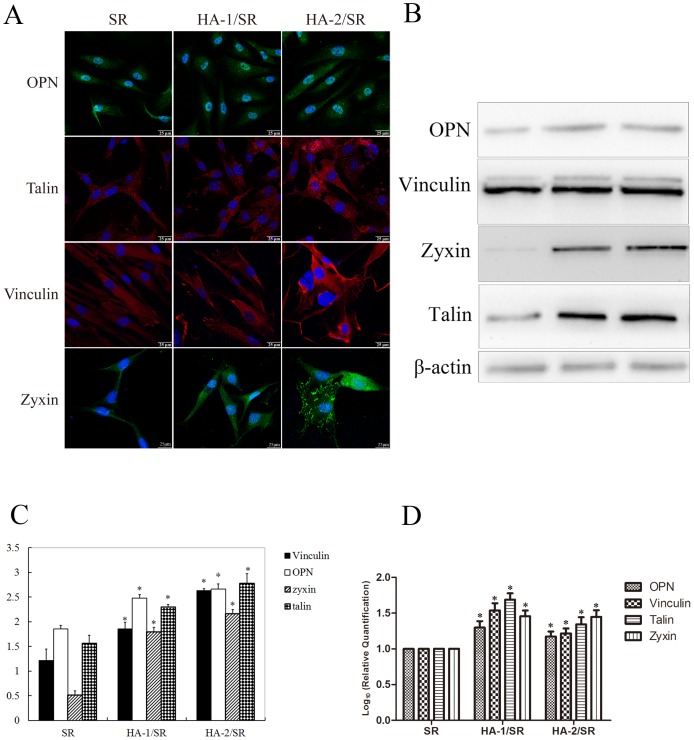
Detection of cell adhesion molecules. A, Immuno-fluorescence of the cells on the three kinds of SR (the scale bar is 20 µm); B, Western-blot of the cells on the three kinds of SR; C, Optical density ratio of Western-blot; D, Realtime PCR of the cells on the three kinds of SR (n = 6, *p<0.05).

Human dermal fibroblast cells were cultured on the surface of the SR, HA-1/SR, and HA-2/SR for 48 h, total cellular protein was extracted. Western blot analysis of the cell extracts that there were significant vinculin expression changes among the HA-1/SR and HA-2/SR groups compared with the SR group (*p*<0.05). The HA-1/SR and HA-2/SR groups showed greater expression, which may also indicate that the cell adhesion had improved with HA coating. Osteopontin (OPN) protein expression of the HA-1/SR and HA-2/SR groups was also significantly stronger than in the SR group (*p*<0.05). These results collectively indicate that without HA coating, SR is a poorer substrate for cells than with the HA coating SR, which improved the SR material properties to the extent that it became a better surface to support cell adhesion. In addition, zyxin and talin were also selected as candidate proteins; their protein expression levels were also greater in the HA-coated groups than in the noncoated group, the results both in Western-blot and realtime PCR are all the same (Figure 3C).

### Mass spectrometry analysis of cellular protein expression on the surface of HA coated SR

**Figure 4:**
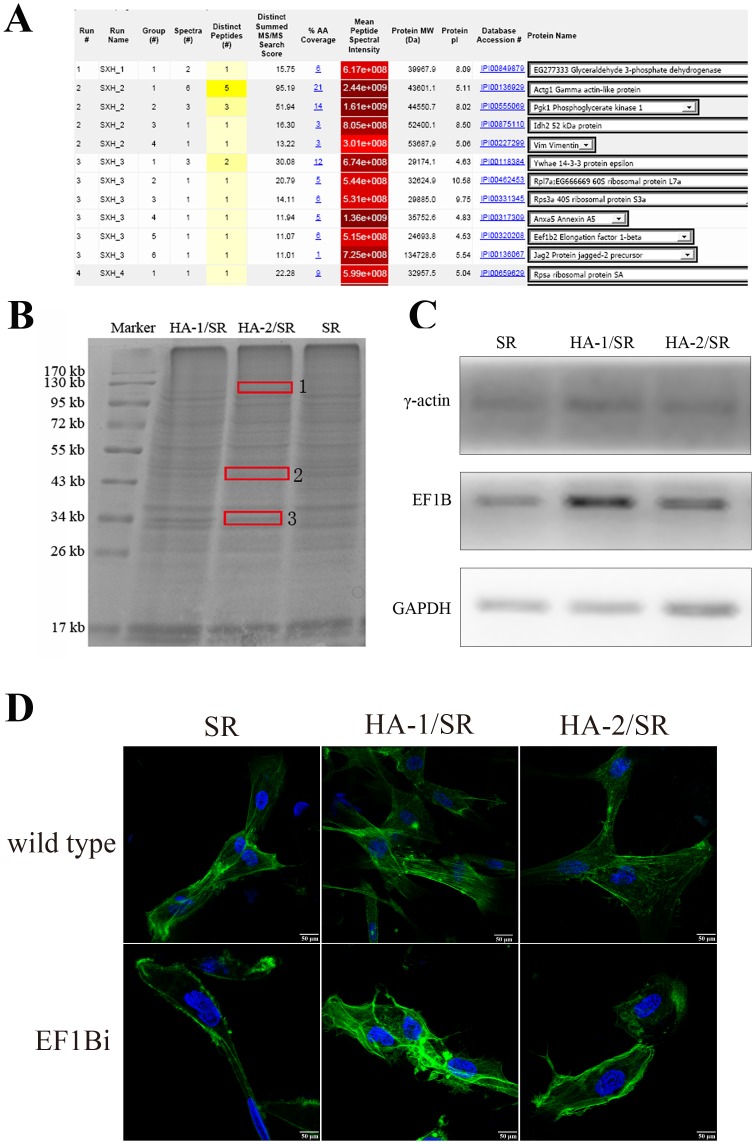
The mechanism of improved cell adhesion on the HA coated surface of SR. A, mass result; B, SDS-PAGE picture (1, 2, 3 represent the selected protein points); C, western-blot verification; D, Cell morphology of EF1β interference human dermal fibroblasts on the three kinds of SR (the scale bar is 50 µm) (wild type means common human dermal fibroblasts, EF1Bi means EF1β interference human dermal fibroblasts).

As the results presented above, the compatibility to cells imparted by HA coating to SR implant materials was significantly increased. To better understand the signaling mechanisms behind this phenomenon, the cells cultured on the surface of the three kinds of SR were harvested after 48 h and total protein was extracted. Protein bands were isolated and identified by mass spectrometry. As shown in Figure 4B, the total protein SDS-PAGE points of difference 1, 2, and 3 were selected and identified by mass spectrometry. The results were analyzed on the website of SRS@EMBL-EBI. According to the references and bioinformatics analysis, elongation factor 1-beta (EF1β) may play an important role in cell adhesion. Furthermore, we hypothesized that EF1β may mediate γ-actin gene expression, leading to cytoskeletal rearrangements. To test this hypothesis, anti EF1β and anti γ-actin IgG were used for western blot analysis (shown in Figure 4C) to analyze EF1β and γ-actin expression levels. The results showed that EF1β expression was greater and γ-actin expression was less in the HA-coated SR compared to the noncoated SR, thus confirming our conjecture. We further analyzed the veracity of this hypothesis by using bioinformatics via the websites of EMBL, Genebank, protsite Documentation etc. We found that EF1β may regulate γ-actin rearrangements and induce increased cell adhesion. In order to confirm this speculation, we reduced the expression of EF1B by RNA interference, found that cytoskeleton in arrangement and the morphology of the cells on the surface of silicone rubber and HA/SR changed, and control to EF1B normal expression, cytoskeleton staining is more shallow, cell area decreased, and cell adhesion decreased (Figure 4D). This confirms that EF1β may regulate γ-actin, affect cytoskeleton arrangement and affect cell adhesion on material surface.

## Discussion

SR is commonly used in plastic surgery. The molecular structure of SR is (SiOR_2_)_n_, in the absence of a double bond; it has stable physical and chemical properties while maintaining good flexibility. Because of its spatial configuration, SR has a relatively low intermolecular force, so it can easily be processed and shaped. Since the SR surface has strong hydrophobicity, it is a poor substrate for cells adhesion. Previous investigators have carried out surface modification or copolymerization with other substances and have achieved remarkable results [4], [21].

HA, being chemically similar to the inorganic component of bone is one of the most popularly used bioactive ceramics in the surgical repair of hard tissue trauma and disease. Successful applications of HA have been witnessed in a range of surgical specialties: bone substitute in bony defects restoration in orthopedic surgery [22], sinus obliteration [23], and ossicular chain reconstruction in otolaryngological surgery, and craniofacial augmentation in plastic surgery [24]. However, HA is fragile and with a low mechanical property witch hamper it clinic using. So coating HA onto the surface of SR can assure the advantage of these two materials and improve the clinic using.

Currently, the common coating methods are aerosol deposition[25], laser cladding [26], plasma spraying [15], and sol –gel [26] methods etc.. Although plasma spraying of metal materials is commonly used, in our study, it has been proven to be difficult to apply on SR. The main reason might be the elastic and soft properties of SR, which surface is difficult for HA plasma spraying coating. Electrochemical reaction pulsed-laser-deposited HA coatings, which are usually used in alloy substrates, have been proven stable and can maintain the bioactivity of HA. But this method requires special equipment and the substrate must be heated to almost 600°C, under which SR will burn. In this study, we coated SR with HA via simple spray painting equipment at the vulcanization stage, and the surface coating was verified by SEM observation. The adhesion of HA to SR is tightly, which was confirmed by surface mechanical challenge (adhesive tape) experiments.

In this research, compare to the mechanical properties such as the Shore hardness, tensile strength, and elongation at breakage of HA-SR decreased. Even so, the HA-coated SR can still meet clinical demands as the Shore hardness ranges from 25 to 35 units in ideal filling materials [27]. The coating materials of HA-1/SR and HA-2/SR had Shore hardness values of 28.14±1.83 and 26.85±1.26 respectively, which are both in line with the soft tissue filling material hardness requirements. According to clinical experience, HA-1/SR and HA-2/SR can be used in the clinic as a soft tissue filling material [28].

Through FTIR analysis, the physical and chemical structures of pure SR, HA-1/SR, and HA-2/SR are similar. From XPS results, we can observe that HA coating significantly changed the surface elemental composition; Ca and P were detected on the surface of HA-1/SR and HA-2/SR, revealing that HA is attached on the SR, possibly facilitating cell adhesion.

In the present study, the dermal fibroblasts cultured on HA/SR grew faster and had a higher mobility and better viability than those cultured on pure SR. The cytoskeleton alignment was improved, and we observed increased expressions of adhesion-associated proteins, including talin-1, zyxin and vinculin in fibroblasts cultured on HA/SR, compared to those on SR. As is known, these proteins are involved in the formation of focal adhesion complexes, acting as a conjugation site for both cytoskeletal organization and intracellular signaling transduction, playing a vital role for cell adhesion and migration. The results indicated that the cell adhesion on HA/SR was significantly improved.

The roughness of materials surface can influence cell adhesion. We selected different particle sizes of hydroxyapatite to coat the surface of SR to prepare HA-coated SR of different roughnesses. To determine if two different particle sizes showed a significant difference, we used HA coating with 40 and 150 µm diameters because they were the only sizes available from the Materials College of Sichuan University. Through the roughness measurement of material surface we found material surface roughness between SR and HA/SR, and the two kinds of HA/SR has remarkable difference. However, using atomic force microscope we did not observed significant difference among the materials surface, the reason maybe that atomic force microscope detection range is too small to detect the difference. Between the two kinds of HA/SR, although the surface roughness is different, but there is no significant difference cytological behavior difference.

In the study presented here, it was interesting to find increased expression of OPN, which is thought to be an important extracellular matrix protein, mediates cell adhesion onto the surface of implants [20]. OPN exists both as a component of the extracellular matrix and as a soluble cytokine, interacting with cells by binding multiple integrins via two major domains that are conserved among species. The ligand-receptor interaction forms a focal adhesion complex, activating signal transduction and provoking recombination of the cytoskeleton. What is known is that this process involves adhesion-associated proteins including talin-1, vinculin, zyxin and others, but the molecular mechanism is still not well understand.

In order to clarify the intracellular cytoskeleton organization and the corresponding regulation mechanism, we utilized gel electrophoresis (SDS-PAGE) and mass spectrometry analysis to identify the proteins that associated with the HA coating. This approach allowed the identification of γ-actin as the major cytoskeletal protein that was expressed at a higher level by dermal fibroblasts cultured on HA-SR, compared with SR. In vertebrates, three main groups of actin isoforms, alpha, beta, and gamma, have been identified. The beta and gamma actins coexist in most cell types as components of the cytoskeleton and mediators of internal cell motility. Whereas the alpha actins are a major constituent of the contractile apparatus, mostly found in muscle tissues. Recent studies have shown that the interaction among adhesion-associated proteins and these proteins are essential to connect extracellular matrix-bound integrins to the cytoskeleton. For example, talin can bind to the cytoskeleton either directly, through its actin-binding motifs, or indirectly, by recruiting other actin-binding proteins [29]. In the current study, we found a significantly increased expression level of γ-actin in HA-coated SR compared to SR, as well as adhesion-associated proteins, including talin-1, vinculin and zyxin, which suggesting that γ-actin is involved in the cytoskeletal rearrangement that regulated by adhesion-associated proteins.

Moreover, we found that eEF1Bα was expressed to a higher level by dermal fibroblasts cultured on HA-SR compared to on SR. The eEF1Bα protein is highly conserved, and has been shown to support the canonical function of GTP/GDP exchange on the eEF1A protein [30]. In addition, eEF1Bα is essential for cellular growth and plays a critical role in translational fidelity [31], [32]. More importantly, eEF1Bα has been proposed as a regulator of eEF1A dependent actin bundling [33]. In the presence of eEF1Bα, eEF1A may lose its ability to bind and cross-link F-actin in vitro. Mutant eEF1Bα, which weakly binds to eEF1A, exhibits remarkable changes in cellular morphology and F-actin organization [33]. In a previous study, eEF1Bα has also been implicated in directly binding to actin and exhibited a concentration-dependent negative effect on actin assembly [34].

In summary, we found significantly increased expression levels of eEF1Bα, γ-actin, and adhesion-associated proteins along with enhanced cell adhesion in HA-coated SR compared to noncoated SR. These findings suggest that improved cell adhesion by HA coating on SR may be mediated by eEF1Bα and γ-actin. However, the challenges that remains to determine the mechanism underlying these phenomenons.
